# Iron-Induced Thrombocytopenia: A Mini-Review of the Literature and Suggested Mechanisms

**DOI:** 10.7759/cureus.10201

**Published:** 2020-09-02

**Authors:** Mona Babikir, Rita Ahmad, Ashraf Soliman, Mustafa Al-Tikrity, Mohamed A Yassin

**Affiliations:** 1 Internal Medicine, Hamad Medical Corporation, Doha, QAT; 2 Family Medicine, Hamad Medical Corporation, Doha, QAT; 3 Pediatric Endocrinology, Hamad Medical Corporation, Doha, QAT; 4 Hematology and Oncology, Hamad General Hospital, Doha, QAT

**Keywords:** deficiency, iron, replacement, supplement, anemia, thrombocytopenia, platelet

## Abstract

Anemia constitutes a major global health burden, and iron deficiency is the most common cause of it. Iron deficiency and replacement affect not only hemoglobin (Hb) levels but also other hematological parameters such as platelet count. In this mini-review, we explore thrombocytopenia as a side effect of iron replacement therapy. We searched for relevant articles published in English, and all case reports/series of iron-induced thrombocytopenia were collected and analyzed. A total of 11 case reports and one case series were found relating to very low Hb at a baseline level of 5.25 +/- 2.2 g/dl and variable platelet count at baseline that dropped in 9 +/-3 days to an average of 121 +/- 112 x 10^9^/L, which in most of the cases was self-corrected. The parenteral route was more commonly reported to be associated with thrombocytopenia, and discontinuation of therapy was needed in two patients.

The mechanisms, prevalence, and clinical significance of thrombocytopenia associated with iron replacement are unknown; several effects of iron on the primary hematopoietic cells and stromal cell lines have been proposed, such as influence on common progenitors, effects on cytokines, and thrombopoietic effect of erythropoietin, which is directly affected by iron levels. Iron replacement can lead to significant thrombocytopenia. Further research is needed to describe the exact incidence, mechanism, and clinical significance of thrombocytopenia associated with iron supplementation.

## Introduction and background

Anemia is defined by the World Health Organization as “a condition in which the number of red blood cells (and consequently their oxygen-carrying capacity) is insufficient to meet the body’s physiologic needs” [1]. The worldwide prevalence of anemia at ∼24.8% [2] and its association with increased morbidity and mortality and decreased work productivity have made it an important public health burden [1,2]. Iron deficiency is the most common cause of anemia worldwide. Approximately 5% and 2% of American women and men, respectively, suffer from iron deficiency anemia (IDA) [3]. It occurs due to the unmet demand for iron to form normal red blood cells.

IDA is generally caused by inadequate intake of iron, chronic blood loss, or a combination of both [3-5]. A less common cause is malabsorption of iron secondary to gastric and/or duodenal resection or inflammatory conditions like infectious or atrophic gastritis, or tropical sprue. A rare cause includes a genetic mutation in TMPRSS6 leading to iron-refractory IDA [6]. Iron deficiency is often associated with thrombocytosis rather than thrombocytopenia; however, both have been described in the literature for more than 50 years [7]. On the other hand, iron replacement has a similar range of effects on thrombopoiesis [8].

In this article, we review the relevant literature published in English to shed light on the link between iron replacement and thrombocytopenia in patients with IDA.

## Review

Methods

We searched the databases PubMed and Scopus for all relevant articles published in the literature in English using the following Medical Subject Headings (MeSH) terms: [deficiency, Iron] [replacement] [supplement] [anemia] [thrombocytopenia] [platelet]. Data were then collected, recorded, and analyzed.

Results

A total of 11 individual case reports and one case series (containing 9 patients) were found, which are summarized in Tables 1-4 [9-19]. The age of the patients in the study ranged between 15 and 42 years, and a female predominance was observed (a female-to-male ratio of approximately 3:1) (Table 1). At baseline, the hemoglobin (Hb) concentration was 5.25 +/- 2.2 g/dl, serum iron concentration was 20 +/- 12.8 ug/dl, and serum ferritin level was 9 +/- 11.8 ng/dl, denoting a severe form of IDA. After iron replacement, Hb concentration increased to 8.03 +/- 1.47 and serum iron concentration increased to 78 +/- 22.5 ug/dl (Table 2). Before iron therapy, the platelet count was 469 +/- 404 x 10^9^/L. The nadir platelet count occurred after 9 +/- 3 days to a mean count of 121 +/- 112 x 10^9^/L (Table 3). The intravenous route was associated with a higher incidence of iron-associated thrombocytopenia than oral and intramuscular routes. Two patients needed platelet transfusions. Thrombocytopenia led to discontinuation of iron replacement in two patients. Both were receiving iron sucrose (Table 4). In the last follow-up of those patients, Hb concentration was found to be increased to 11.5 +/- 2.3 g/dl, and platelet count had increased to 322 +/- 233 x 10^9^/L.

**Table 1:** Cases publication year, and age, gender, and ethnicity of patients NA: not available

No	Authors	Year of publication	Patient gender	Patient age (years)	Patient ethnicity
1	Giordano et al. [9]	2019	Male	16	African
2	Bayhan et al. [10]	2016	Female	15	NA
3	Kahraman et al. [11]	2016	Male	17	NA
4	Cunha et al. [12]	2015	Female	16	NA
5	Lindgren et al. [13]	2009	Male	NA	NA
6	Ganti et al. [14]	2007	Female	39	African American
7	Taskapan et al. [15]	2003	Female	37	NA
8	Go et al. [16]	2000	Female	30	NA
9	Soff and Levin (case 1) [17]	1988	Female	17	NA
10	Soff and Levin (case 2) [17]	1988	Female	17	NA
11	Knizley et al. [18]	1972	Male	42	Black
12	Lioger et al. (series) [19]	2016	1 male, 8 females	NA	NA

**Table 2:** Hemoglobin and iron profile at baseline and during and after iron therapy Hb: hemoglobin; MCV: mean corpuscular volume; Fe sat: iron saturation; NA: not available

No	Authors	Hb at baseline	MCV	Hb during replacement	Hb after replacement	Iron at baseline	Iron after replacement	Fe sat at baseline	Fe sat after replacement	Ferritin at baseline	Ferritin after replacement
1	Giordano et al. [9]	5.2 g/dL	54.5 fL	8.4 g/dL	13.3 g/dL	NA	NA	NA	NA	3.1 ng/mL	NA
2	Bayhan et al. [10]	4.7 g/dL	64.2 fL	8.2 g/dL	9.6 g/dL	NA	NA	9.50%	Na	2.4 ng/mL	NA
3	Kahraman et al. [11]	7.6 g/dL	66 fL	NA	NA	27 μg/dL	NA	NA	NA	3 ng/mL	NA
4	Cunha et al. [12]	5.8 g/dL	63 fL	8.1 g/dl	8.8 g/dl	NA	78 mcg/dl	NA	NA	NA	NA
5	Lindgren et al. [13]	NA	NA	NA	NA	NA	NA	NA	NA	NA	NA
6	Ganti et al. [14]	3.1 g/dL	58.6 fL	5.2 g/dL	14.0 g/dL	17 µg/dL	NA	4%	NA	<2 ng/dL	121 ng/mL
7	Taskapan et al. [15]	10.1 g/dL	88.2 fL	8.9 g/dL	9.9 g/dL	19 μg/dL	NA	7%	NA	35.5 µg/L	NA
8	Go et al. [16]	3.1 g/dL	77 fL	NA	NA	5 µ/dL	NA	1%	NA	8 mg/L	NA
9	Soff and Levin (case 1) [17]	3.2 g/dL				22 µg/dL	NA	NA	NA	<10 ng/ml	NA
10	Soff and Levin (case 1) [17]	3.2 g/dL	Low	NA	NA	7 µ/dL	NA	NA	NA	NA	NA
11	Knizley et al. [18]	6.1 g/dL		9.4 g/dL	13.6 g/dL	43 mg/dl	NA	8%	NA	NA	NA
12	Lioger et al. (series) [19]	5.7 ± 2.3 g/dL	63 fL	NA	NA	NA	NA	NA	NA	NA	

**Table 3:** Platelet count before, during, and after replacement NA: not available

No	Authors	Platelet at baseline	Lowest platelet count	Days to nadir	Last platelet count
1	Giordano et al. [9]	40 k/μL	28 k/μL	5	169 k/μL
2	Bayhan et al. [10]	820 x 10^9^/L	48 x 10^9^/L	12	363 x 10^9^/L
3	Kahraman et al. [11]	340 x 10^3^/mcL	68 x 10^3^/mcL	7	159 x 10^3^/mcL
4	Cunha et al. [12]	424 x 10^9^/L	45 x 10^9^/L	8	666 x 10^9^/L
5	Lindgren et al. [13]	NA	NA	NA	NA
6	Ganti et al. [14]	127 x 10^3^/mcL	39 x 10^3^/mcL	2	181 x 10^3^/mcL
7	Taskapan et al. [15]	102,000/L	2,000/L	8	87,000/L
8	Go et al. [16]	426 x 10^9^/L	20 x 10^9^/L	8	NA
9	Soff and Levin (case 1) [17]	168,000/mm^3^	21,000/mm^3^	6	683,000/mm^3^
10	Soff and Levin (case 2) [17]	717,000/mm^3^	105,000/mm^3^	10	NA
11	Knizley et al. [18]	1,465,000/mm^3^	37,000/mm^3^	13	265,000/mm^3^
12	Lioger et al. (series) [19]	172 ± 133 x 10^9^/L (range: 102-434)	85% from baseline		NA

**Table 4:** Iron replacement route, preparation, and dose, and whether discontinuation/transfusion was required IV: intravenous; IM: intramuscular; NA: not available

No	Authors	Route of iron replacement	Type of iron replacement	Dose	Discontinuation
1	Giordano et al. [9]	Oral	Ferrous sulfate	NA	No
2	Bayhan et al. [10]	Oral	Ferrous glycine sulfate	4 mg/kg	No
3	Kahraman et al. [11]	IV	Iron sucrose	100 mg/day	No
4	Cunha et al. [12]	IV	Iron sucrose	100 mg	Yes
5	Lindgren et al. [13]	NA	NA	NA	NA
6	Ganti et al. [14]	IV	Iron sucrose	100 mg 2xwk	No
7	Taskapan et al. [15]	IV	Iron sucrose	200 mg daily	No
8	Go et al. [16]	IM	Iron dextran	100 mg/day	Yes (changed to oral)
9	Soff and Levin (case 1) [17]	Oral	Ferrous sulfate	300 mg TID	NA
10	Soff and Levin (case 2) [17]	Oral	Ferrous gluconate		NA
11	Knizley et al. [18]	Oral	Ferrous sulfate	235 mg TID	No
12	Lioger et al. (series) [19]	IV: 8, IM: 1	Iron sucrose/ferric carboxymaltose	NA	NA

Discussion

Thrombocytopenia may occur in patients with severe IDA who receive iron therapy; however, it is very uncommon [3]. Its pathogenesis remains undetermined but is thought to be related to the alteration in the activity of iron-dependent enzymes involved in thrombopoiesis [14,20].

Iron has a synthetic and regulatory role in platelet production, and animal models have suggested a common progenitor cell for erythroid and megakaryocytic cell lineages [21]. The reviewed cases who developed thrombocytopenia after iron replacement, especially parenteral iron, had a significant reduction in platelet count that was self-corrected in most of the cases. The mechanisms, prevalence, and clinical significance of this thrombocytopenia are unknown [15-18,22]. Iron has a synthetic and regulatory role in platelet production. Iron can induce cell death by generating free radicals as it interconverts between ferrous (Fe2+) and ferric (Fe3+) forms. A randomized, placebo-controlled, double-blind, parallel-group study was performed on 38 peritoneal dialysis patients before and after a single intravenous infusion of 300 mg iron sucrose. Iron infusion increased in total (Δ 601 μg/100 mL, CI 507, 696) and non-transferrin-bound iron (Δ 237.2 μmol/L, CI 173.6, 300.8) increased approximately by 10-fold, as well as redox-active iron by nearly five-fold (Δ 0.76 μmol/L, CI 0.54, 0.98) [23].

Animal models have suggested a common progenitor cell for erythroid and megakaryocytic cell lineages [21]. An in vitro study examined the effects of iron load (that may simulate the process of iron infusion in patients with IDA) on the function of primary hematopoietic cells and stromal cell lines and found that iron overload impairs normal hematopoietic cells and modifies the function of stromal cells, indicating that iron overload impairs the whole hematopoietic system. The authors suggested that the deleterious effects of iron were mediated through its impact on hematopoietic stem/progenitor cells as well as in differentiated hematopoietic via reactive oxygen species (ROS) accumulation [24]. In addition, an in-vitro study showed that ferrous ammonium sulfate (FeAS) induced growth arrest and apoptosis in immature hematopoietic cells and led to insulin-like growth factor binding protein 2 (IGFBP2) and insulin-like growth factor 1 (IGF-1) down-regulation. Both have been found to stimulate the survival, proliferation, and cycling of hematopoietic stem cells (HSCs) [25].

Although thrombopoietin is the primary growth factor and regulator of megakaryopoiesis, several other cytokines such as interleukin (IL)-1, IL-3, IL-6, IL-11, and tumor necrosis factor are also involved in this process [26-28]. Erythropoietin shares some structural features with thrombopoietin and may exhibit a synergistic effect on platelet production. The levels of endogenous erythropoietin significantly decrease during the correction of iron deficiency. This reduction in endogenous erythropoietin might decrease the productivity of megakaryocytes and lead to transient thrombocytopenia [29,30].

In this study, we observed that young females are more likely to be affected by IDA. Further research is needed to explore whether this is because IDA per se is more common in this population or if there are other explanations related to female sex hormone or genetic profile. Another observation was that platelet nadir occurred within 13 days of iron replacement initiation; however, the number of cases was too small to recommend following platelet count in asymptomatic patients within this timeframe.

## Conclusions

Significant thrombocytopenia occasionally occurs in patients receiving iron therapy for IDA. This condition is transient and self-limited in most of these cases. Various mechanisms can contribute to this thrombocytopenia. Dedicated observational and experimental studies are needed to describe the correlation between iron profile, hemoglobin level, genetic factors, cytokines, growth factors and thrombopoiesis; these studies should look for predictors that could determine which individuals will get thrombocytopenia on receiving iron supplements.
